# Fluoride Concentration of Bottled Water and Tap Water in Tehran, Iran

**DOI:** 10.5681/joddd.2011.030

**Published:** 2011-12-19

**Authors:** Masoumeh Moslemi, Zahra Khalili, Soraya Karimi, Mohammad Mostafa Shadkar

**Affiliations:** ^1^Professor, Department of Pediatric Dentistry, Faculty of Dentistry, Shahid Beheshti University of Medical Sciences, Tehran, Iran; ^2^Assistant Professor, Department of Pediatric Dentistry, Faculty of Dentistry, Shahed University of Medical Sciences, Tehran, Iran; ^3^Dentist, Private Practice, Tehran, Iran; ^4^Dentist, Faculty of Dentistry, Shahid Beheshti University of Medical Sciences, Tehran, Iran

**Keywords:** Caries control, drinking water, fluoride, water

## Abstract

**Background and aims:**

As a result of poor quality of public water supply in many countries, people have recently turned to bottled water consumption, the fluoride content of which is not generally consistent among different brands. This study sought to measure the fluoride concentration of public water supply in comparison with commercial brands of min-eral bottled water available in Tehran, Iran.

**Materials and methods:**

Eight different brands of locally produced bottled mineral water and samples of tap water were evaluated for fluoride content. All samples were collected in five equal containers in two summer and winter seasons. The fluoride content in part per million (ppm) was determined using a fluoride Ion Selective Electrode. The data were ana-lyzed using two-way ANOVA. For comparison of the fluoride content between three different brands of bottled water, one-way ANOVA was employed. Sample t-test was used to compare the label and laboratory values of bottled water.

**Results:**

The highest concentration of fluoride in a bottled water brand was found to be 0.409 ppm with a pH of 6.67 in summer. There was a significant difference between the mean fluoride level of tap water (0.229 ± 0.079 ppm) and bottled water (0.111 ± 0.122 ppm) (P < 0.001). The measured fluoride concentrations of bottled water were significantly lower than those printed on the labels (P < 0.001).

**Conclusion:**

Our findings revealed that the mean fluoride level of both bottled and tap water samples evaluated is con-siderably lower than accepted standards.

## Introduction

Fluoride has been considered a valuable source of oral health promotion in children and adults over the past fifty years. The primary mode of action of fluoride in reducing dental caries is that it promotes remineralization and inhibits demineralization of tooth structure, predominantly effective in restraining incipient caries. Therefore, post-eruptive absorption of fluoride throughout one’s life should be emphasized. Adjustment of daily fluoride intake should be taken with special considerations since a low amount of fluoride is not effective in preventing dental caries, and higher concentrations lead to dental fluorosis and, in more severe cases, may have adverse effects on bones, the kidneys, and the thyroid gland. Hence, it is crucial that oral health practitioners monitor the amount of fluoride intake, especially in children. ^1 - 3^

Water fluoridation is considered as one of the most efficient methods in reducing dental caries on a public health level and has its greatest influence on socially disadvantaged children with higher prevalence of tooth decay.^4 - 6^A systematic review provided strong evidence that water fluoridation was effective in reducing overall dental caries in communities.^7^ The U.S. Centers for Disease Control listed water fluoridation as one of the ten great public health achievements of the twentieth century.^8^

A common trend in recent years has been the replacement of purified tap water for drinking with bottled water.^9^ Different studies have reported inconsistency between the actual fluoride content of bottled water and the amount mentioned on its container label.^2 , 10 , 11^

Although countries have close surveillance on the manufacturers of bottled water regarding the concentrations of different elements, there is evidence supporting the inaccuracy of the reported fluoride content of tap and bottled water.^12^

The present study aimed to evaluate the fluoride content of public tap water as well as available commercial brands of bottled water in Tehran, Iran.

## Materials and Methods

Eight commercially available brands of locally produced bottled mineral water were randomly selected and considered for fluoride content evaluation. Samples of tap water were also collected from different residential areas of Tehran, representing the four main water plants i.e. Karaj dam, Latian dam, and Lar dam. Samples were collected in five equal containers in two different seasons (summer and winter). All samples were stored sealed in their original containers until the fluoride analysis was performed. After shaking the sample container, 1 mL was taken and mixed with 0.1 mL of Total Ionic Strength Adjusting Buffer III (TISAB II, Orion, MA, USA). The fluoride concentrations of all 125 samples were determined using Fluoride Ion Selective Electrode (model 96-09, ATI Orion) in conjunction with an ISE Meter (Model 720A, ATI Orion). Fluoride standards ranging from 0.001 to 10.00 mgL ^-1^ were used to calibrate the measurement. The pH of the water samples was also measured using a pH-meter (Model 240, Corning). 10 samples were randomly selected and re-analyzed to assess the reliability of the method. All samples were number coded so that the investigators were blind to the type of water contained in the bottles.

In order to compare fluoride content of tap water with bottled water in two different seasons, the data were analyzed using two-way ANOVA. For comparison of the fluoride content between three different brands of bottled water, one-way ANOVA was employed. Sample t-test was used to compare the label and laboratory values of bottled water. All measurements were analyzed using SPSS 15.0.

## Results

Table 1 demonstrates the mean fluoride content and the pH values of the samples. The lowest fluoride concentration for bottled mineral water collected in summer was 0.009 ppm with a pH of 7.54 and the highest concentration was 0.409 ppm with a pH of 6.67. Among the bottled water samples collected in winter, the lowest and the highest fluoride contents were seen to be 0.002 ppm and 0.387ppm, respectively. The results also indicated that of the water plants supplying the tap drinking water of Tehran, Karaj dam contains the lowest concentration of fluoride during winter (0.129 ppm) and Lar dam contains the highest concentration of fluoride during summer (0.358 ppm).

**Table 1:** Fluoride concentrations of eight brands of bottled waters and tap water from three water plants and pH values

	Summer		Winter		
Type of water	F Concentration (PPM)	pH	F Concentration (PPM)	pH	Labeled content
Bottled mineral water					
Kouhrang	0.210	7.55	0.190	7.60	0.23
Vata	0.090	7.30	0.057	7.24	0.11
Pure Life	0.068	8.00	0.045	8.02	0.07
Dessani	0.409	6.67	0.387	6.54	0.6–1.1
Zamzam	0.040	6.09	0.028	6.70	Not labeled
Bisheh	0.037	7.47	0.022	7.39	0.07
Damavand	0.107	6.66	0.083	6.53	0.2
Damash	0.009	7.54	0.002	7.59	0.2>
Tap water					
Karaj dam	0.182	7.58	0.129	7.43	N/A
Latian dam	0.208	7.54	0.194	7.44	N/A
Lar dam	0.358	7.62	0.305	7.58	N/A

The two-way ANOVA test results failed to show significant difference between fluoride concentrations in water samples collected over summer and winter (P = 0.22). However, there was a significant difference between fluoride content of mineral bottled water and tap water (P < 0.001) (Table 2).

**Table 2:** Fluoride concentration of bottled mineral and tap water samples by seasonal difference

Type of water	Number	Mean F Level (SEM)
Summer		
Bottled Water	40	0.101 ± 0.019
Tap Water	15	0.209 ± 0.019
Total	55	0.131 ± 0.016
Winter		
Bottled Water	40	0.121 ± 0.020
Tap Water	15	0.249 ± 0.021
Total	55	0.156 ± 0.017
Total		
Bottled Water	80	0.111±0.014
Tap Water	30	0.229 ± 0.014
Total	110	0.143 ± 0.012

Further analysis comparing the fluoride concentration among three randomly chosen brands of bottled mineral water, documented significant difference between the three brands (highest mean fluoride, 0.196 ± 0.004 ppm; lowest mean fluoride, 0.052 ± 0.001 ppm; P < 0.001; Table 3).

**Table 3:** Fluoride level of three randomly selected bottled mineral water

Brand	Number	Mean F Level (SEM)	pH	Labeled content
Kouhrang	5	0.196 ± 0.002	7.59	0.23
Vata	5	0.083 ± 0.000	7.31	0.11
Purelife	5	0.052 ± 0.000	7.90	0.07

The results indicated that the claimed fluoride concentrations on the labels were significantly above the levels measured in this study (P < 0.001).

## Discussion

Fluoridated water is generally regarded as a safe and accessible way to prevent dental caries among all groups of the society.^13^ In view of the fact that the fluoride content of tap water is reported inconsistently throughout the country and there is an overgrowing trend toward the consumption of bottled mineral water, it is essential to control the concentration of different elements including fluoride in these products.

In order to measure the fluoride concentration, we employed Ion Selective Electrode method as a reliable device previously utilized by Zohouri et al. and Cochrane et al.^12 , 14^ However, other investigators applied spectrophotometer which is said to be prone to potential errors.^15 , 16^

In this study, we attempted to measure the fluoride concentration of tap and bottled mineral water as two major sources of fluoride, which, to the best of our knowledge, is only similar to a previous study by Hurtado & Gardea-Torresday.^17^ In most of other studies, the investigators have only assessed the fluoride content in either one of the two water supplies.

In order to assess the role of seasonal variations in the concentration of fluoride, we collected our samples in two different seasons, i.e. summer and winter. Our findings confirmed those of previous studies reporting that higher fluoride concentration is associated with warm and dry climates.^18 , 19^ This could be related to the chemical reactions of underground waters with volcanic products. However, statistical analysis did not reveal any significant difference in this regard.

The finding that the actual fluoride levels were significantly lower than those displayed on the labels confirms previous reports suggesting that the manufacturers need to enhance their commitment to high standards and pay more attention to the figures stated on their products.^9 , 12 , 16^

The fluoride concentration of both bottled mineral water and tap water samples in the present study was measured below the standard level. This might be attributed to the type of local soil and its mineral content, seasonal variations and possible presence of volcanic sediments in underground water reservoirs.^14 , 16 , 19 , 20^

## Conclusion

The results indicate that the fluoride content of mineral bottled water and local tap water in Tehran is lower than the optimal level. Moreover, mean concentration of fluoride in public tap water is significantly higher than mineral bottled water. The actual fluoride content of bottled waters was significantly lower than the figures on their labels.
